# HbA1c Cutoff Point of 5.9% Better Identifies High Risk of Progression to Diabetes among Chinese Adults: Results from a Retrospective Cohort Study

**DOI:** 10.1155/2018/7486493

**Published:** 2018-06-13

**Authors:** Kai Liang, Chuan Wang, Fei Yan, Lingshu Wang, Tianyi He, Xiuping Zhang, Chengqiao Li, Weifang Yang, Zeqiang Ma, Aixia Ma, Xinguo Hou, Li Chen

**Affiliations:** ^1^Department of Endocrinology, Qilu Hospital of Shandong University, Jinan, China; ^2^Institute of Endocrinology and Metabolism Disease, Shandong University, Jinan, China; ^3^Key Laboratory of Endocrinology and Metabolism Disease, Shandong Province Medicine & Health, Jinan, China; ^4^Shantui Community Health Center, Jining, China; ^5^Department of Endocrinology, Second People's Hospital, Jining, China; ^6^Lukang Hospital, Jining, China; ^7^China National Heavy-Duty Truck Group Corporation Hospital, Jinan, China

## Abstract

**Aims:**

This article performed a retrospective cohort study to estimate the annual incidence rates of diabetes and to assess the utility of HbA1c as a predictor for progression to diabetes in Chinese community adults aged 40 years or older.

**Methods:**

In all, 2778 nondiabetic subjects (including 1901 women) underwent HbA1c testing and oral glucose tolerance test (OGTT) measurements at baseline and after 3 years. Diabetes and prediabetes were defined using the WHO criteria. The HbA1c cutoff points were evaluated to predict the future risks of diabetes. Relative risk (RR) was calculated using the chi-square test. The area under the receiver operating characteristic (ROC) curve (AUC) was used to evaluate the predictive efficiency of fasting plasma glucose (FPG), 2 hr postprandial plasma glucose (2hPG), and HbA1c for progression to diabetes. A superior cutoff point was defined as the point on the ROC curve with a larger Youden index.

**Results:**

Overall, 7.5% (210/2778) of the subjects progressed to diabetes, yielding an annual 2.5% diabetes incidence rate. Additionally, 4.5% (100/2227) of the subjects with normal glucose tolerance (NGT) and 19.6% (110/561) of the subjects with prediabetes progressed to diabetes, and the relative risk of progression to diabetes was 5.188 times higher in subjects with prediabetes than in subjects with NGT (*p* < 0.001). Compared to subjects with HbA1c values ≤ 5.6%, the RRs of progression to diabetes in subjects whose HbA1c ranged from 5.7 to 5.8%, 5.9 to 6.2%, 6.3 to 6.4%, and ≥6.5% were 1.165, 2.582, 5.732, and 16.619, respectively. However, the RRs for subjects with HbA1c ranging from 5.7 to 5.8% and those with HbA1c ≤ 5.6% did not differ significantly (*p* = 0.615). The AUCs for predicting diabetes after 3 years by FPG, 2hPG, and HbA1c were 0.752 (95% confidence interval 0.718–0.787), 0.710 (95% confidence interval 0.671–0.748), and 0.756 (95% confidence interval 0.720–0.793), respectively. The HbA1c cutoff point of 5.9% (sensitivity of 0.771 and specificity of 0.580) may better identify individuals at high risk of progression to diabetes than the 5.7% value (sensitivity of 0.862 and specificity of 0.371) due to the former's larger Youden index of 0.351, which exceeded the indices for FPG and 2hPG.

**Conclusions:**

The use of HbA1c values ≥ 5.9% may provide greater accuracy in evaluating the risk of progression to diabetes and identify individuals with prediabetes with greater reliability among Chinese adults.

## 1. Introduction

According to a recent survey, an estimated 148.2 million Chinese adults have prediabetes [1], which may have serious effects on public health considering their high risk of developing diabetes. However, not all people with prediabetes progress to diabetes, and effective lifestyle interventions are beneficial to prevent the onset of type 2 diabetes or delay its progression [2–4]. Therefore, an indicator used to identify people at highest risk of progression to diabetes should be optimal.

An HbA1c value of ≥6.5% has been adopted for the diagnosis of diabetes according to the clinical practice recommendations from the American Diabetes Association (ADA), the European Association for the Study of Diabetes (EASD), and the International Diabetes Federation (IDF) [5, 6], partly based on the association of HbA1c with retinopathy. However, the use of HbA1c values between 5.7 and 6.4% for diagnosing prediabetes is not widely accepted because the adequacy of HbA1c as a reliable diagnostic tool for prediabetes remains under debate [7–9], and the cutoff points vary by race.

Our previous cross-sectional study proposed an HbA1c cutoff point of 6.3% to diagnose diabetes and 5.9% to diagnose prediabetes in Chinese adults [10]. However, that study provided insufficient evidence regarding the utility of HbA1c to predict future risks of diabetes. In this article, we performed a retrospective cohort study to estimate the annual incidence rate of diabetes and to assess the ability of HbA1c to predict progression to diabetes in Chinese adults aged 40 years or older.

## 2. Materials and Methods

### 2.1. Study Population

The present study was one component of the baseline and 3-year follow-up surveys conducted for the Risk Evaluation of cAncers in Chinese diabeTic Individuals: a lONgitudinal (REACTION) study [11, 12]. In the 2012 baseline survey, we studied 10,028 subjects (including 6458 women) aged 40 to 90 years from 4 urban communities (1 from Jinan City and 3 from Jining City) in Shandong Province, China. During 2015, we performed a 3-year follow-up, which served as the first visit following the baseline survey. That follow-up included 4778 subjects who participated in the on-site follow-up and underwent repeat HbA1c and oral glucose tolerance test (OGTT) measurements, 2864 subjects who received a telephone survey, 159 subjects who did not survive to follow-up, and 2227 subjects who were lost to follow-up, which yielded a follow-up rate of 77.8%. Of the 4778 subjects who participated in the on-site follow-up, we excluded 2000 subjects who had been diagnosed previously with diabetes (*n* = 1073), were newly diagnosed with diabetes at baseline (*n* = 462), had liver dysfunction (*n* = 94), had chronic kidney disease (*n* = 68), had cancer (*n* = 27), had undergone gastrointestinal surgery (*n* = 2), splenectomy (*n* = 1), or glucocorticoid treatment (*n* = 7), or had incomplete survey data (*n* = 266). Ultimately, 2778 nondiabetic subjects (including 1901 women) at baseline were eligible for the analysis. The institutional review board at the Department of Endocrinology and Metabolic Disease, Ruijin Hospital, Shanghai Jiaotong University School of Medicine approved the study protocol. All subjects gave informed consent.

### 2.2. Data Collection and Clinical Evaluation

All investigators who participated in both surveys received extensive training related to the study questionnaire and outcome measures before the investigation. A standard questionnaire applied through face-to-face interviews was used to obtain data on demographic characteristics and lifestyle. The anthropometric data collected included height, weight, waist circumference (WC), and blood pressure (BP). Body mass index (BMI) was calculated as weight (kg) divided by height squared (m^2^). WC was measured from the midpoint between the lower borders of the rib cage and the anterior superior iliac spine. Three consecutive BP measurements were obtained at 1 min intervals using the right arm, and the mean of the 3 measurements was used for analysis.

Blood samples were collected in the morning after at least 10 hr of overnight fasting and 2 hr after ingesting a 75 g oral glucose load for the OGTT, which was determined by the glucose oxidase method using an automated clinical chemistry analyzer. HbA1c was determined by ion-exchange high-performance liquid chromatography using an automated glycated hemoglobin meter (VARIANT™, Bio-Rad, USA). All clinical determinations were measured according to the manufacturers' instructions.

### 2.3. Definitions and Diagnostic Criteria

Diabetes and prediabetes were defined using the WHO criteria in this study, rather than ADA guidelines. According to the 1999 World Health Organization diagnostic criteria [13], newly diagnosed diabetes was defined as fasting plasma glucose (FPG) ≥7.0 mmol/L and/or 2 hr postprandial plasma glucose (2hPG) ≥11.1 mmol/L. Prediabetes, also known as impaired glucose regulation (IGR) features isolated impaired fasting glucose (i-IFG), isolated impaired glucose tolerance (i-IGT), and combined impaired glucose tolerance (c-IGT). i-IFG was defined as FPG ≥ 6.1 mmol/L and <7.0 mmol/L, and 2hPG < 7.8 mmol/L. i-IGT was defined as FPG < 6.1 mmol/L, and 2hPG ≥ 7.8 mmol/L and <11.1 mmol/L. c-IGT was defined as FPG ≥ 6.1 mmol/L and <7.0 mmol/L, and 2hPG ≥ 7.8 mmol/L and <11.1 mmol/L. According to the four HbA1c cutoff points, two cutoff points of 5.7% and 6.5% from the ADA guidelines[5] and two cutoff points of 5.9% and 6.3% from our previous study [10], we divided the subjects into the following five groups: ≤5.6%, 5.7–5.8%, 5.9–6.2%, 6.3–6.4%, and ≥6.5%.

### 2.4. Statistical Analysis

Statistical analyses were performed using SPSS 22.0 (SPSS, Chicago, Illinois). Continuous variables with normal distributions were expressed as the mean ± SD, and categorical variables were expressed as the number (proportion). Relative risk (RR) was calculated using the chi-square test. The area under the receiver operating characteristic (ROC) curve (AUC) was determined to evaluate the predictive value of progression to diabetes after 3 years using the baseline blood glucose and HbA1c values. The sensitivity, specificity, positive predictive value, negative predictive value, positive likelihood ratio, and negative likelihood ratio were determined according to the diagnostic testing methodology. The point with the larger Youden index, equal to sensitivity + specificity − 1, was defined as the superior cutoff point. Two-sided values for *p* < 0.05 were considered statistically significant.

## 3. Results

After a median of 3 years, 2778 nondiabetic subjects participated in the 2015 follow-up survey, including 2227 subjects with normal glucose tolerance (NGT) and 561 subjects with IGR. Overall, 7.5% (210/2778) of the subjects progressed to diabetes, which included 4.5% (100/2227) of the subjects with NGT and 19.6% (110/561) of the subjects with IGR. Additionally, 22.8% (508/2227) of the NGT subjects converted to IGR.  Table 1 shows the clinical and biochemical characteristics of the NGT and IGR subjects at baseline based on their glycemic status at follow-up. Although all groups included more females than males, this distribution did not reflect the sex ratio in the population but rather the willingness to participate in the study. Individuals who progressed from NGT to IGR or diabetes were older and had higher BMI, waist circumference, systolic BP, FPG, 2hPG, HbA1c, HDL cholesterol, and serum creatinine values compared to individuals who did not progress. Among the subjects with IGR at baseline, compared to individuals who regressed to NGT or remained as IGR, those who progressed to diabetes had significantly higher FPG, 2hPG, HbA1c, and gamma-glutamyl transferase (GGT) values.

Table 2 shows the diabetes incidence rates among the glucose regulation subgroups after 3 years of follow-up in the study population. Of the 2227 subjects with NGT at baseline, 100 progressed to diabetes for an annual diabetes incidence rate of 1.5%. Among the 561 subjects with IGR at baseline, 110 progressed to diabetes (6.5% annual incidence rate). The relative risk of developing diabetes was 5.188 times higher in subjects with IGR than in NGT subjects (*p* < 0.001). Subjects with combined IGT showed the highest propensity for diabetes. Of greater concern, the incidence rate of diabetes was also estimated by the level of HbA1c at baseline. The RR of developing diabetes in those whose HbA1c values ranged from 5.7 to 5.8% did not differ significantly from that in subjects with HbA1c values ≤ 5.6% (*p* = 0.615). However, in the other three groups whose HbA1c values ranged from 5.9 to 6.2%, 6.3 to 6.4%, and ≥6.5%, the RRs of developing diabetes were significantly higher than those in individuals with HbA1c values ≤ 5.6% (all *p* < 0.001), that is, 2.582, 5.732, and 16.619, respectively. Overall, the annual incidence of diabetes for nondiabetic subjects was 2.5%.

**Figure 1:**
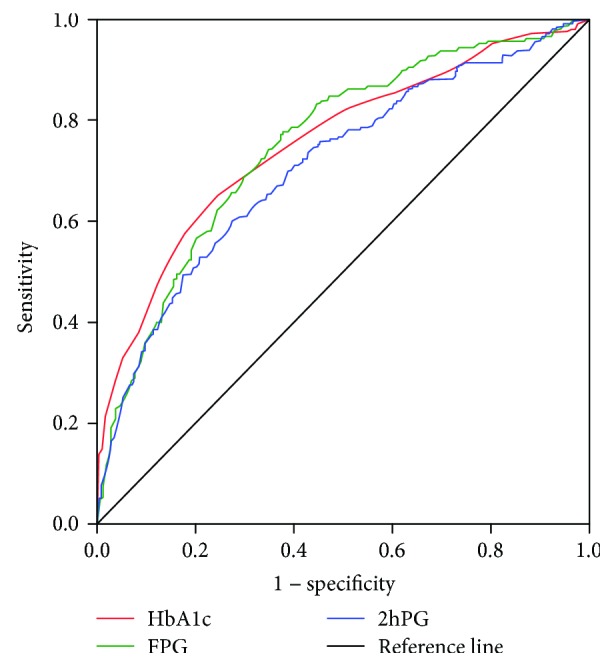
Receiver operating characteristic curve for predicting diabetes after 3 years using baseline blood glucose and HbA1c values. The areas under the ROC curve for FPG, 2hPG, and HbA1c were 0.752 (95% confidence interval 0.718–0.787), 0.710 (95% confidence interval 0.671–0.748), and 0.756 (95% confidence interval 0.720–0.793), respectively.

According to Figure 1, the areas under the ROC curve for predicting progression to diabetes after 3 years by FPG, 2hPG, and HbA1c were 0.752 (95% confidence interval 0.718–0.787), 0.710 (95% confidence interval 0.671–0.748), and 0.756 (95% confidence interval 0.720–0.793), respectively. Therefore, compared with FPG and 2hPG, HbA1c may be a superior indicator for identifying diabetes at the 3-year follow-up.

Table 3 illustrates the predictive value of progression to diabetes after 3 years according to different definitions of IGR at baseline. Both FPG concentration 6.1–6.9 mmol/L (sensitivity of 0.390 and specificity of 0.884) and 2hPG concentration 7.8–11.0 mmol/L (sensitivity of 0.343 and specificity of 0.905) had low sensitivity and high specificity. In contrast, HbA1c 5.7% (sensitivity of 0.862 and specificity of 0.371) and 5.9% (sensitivity of 0.771 and specificity of 0.580) showed high sensitivity and low specificity. Moreover, HbA1c 5.9% may be a preferred cutoff point than 5.7% due to the larger Youden index of 0.351, which exceeded the indices for FPG and 2hPG.

## 4. Discussion

The present study harmonized a baseline survey in 2012 and a 3-year follow-up completed in 2015 among Chinese community adults aged 40–90 years. Of the 2778 nondiabetic subjects at baseline, 7.5% progressed to diabetes, which yielded an annual diabetes incidence rate of 2.5%. The 3-year cumulative incidence rates of diabetes were clearly higher in our study than those in another cross-sectional 3-year follow-up study conducted in China [14] (7.5% versus 4.98%), which might be attributed to the number of subjects aged 40 years or older who participated in the present study. Moreover, the relative risk of progression to diabetes in subjects with IGR was significantly higher than in subjects with NGT. Therefore, early screening and intervention for prediabetes are vital efforts to prevent or delay progression to diabetes [2–4].

Although HbA1c, FPG, and 2hPG values have been recommended as screening tests for prediabetes, the optimal predictor of diabetes development has not been determined thus far, particularly for different races. Additionally, some controversy exists regarding the criteria used to define prediabetes. Currently, the most popular prediabetes diagnostic criteria in China are those of the WHO [13]. Clearly, the ADA FPG cutoff point of 5.6 mmol/L identifies more people with prediabetes than the WHO FPG cutoff point of 6.1 mmol/L. However, the accurate identification of those individuals at highest risk is salient along with the avoidance of inappropriate or excessive diagnosis of these conditions. Therefore, an FPG value of 6.1 mmol/L was used as the cutoff point for prediabetes in this study. Furthermore, although various cutoff levels of HbA1c have been suggested to screen for diabetes, greater consensus regarding the optimal level is required, particularly for different ethnicities [15]. In China, however, HbA1c has not been widely recognized for diagnosing prediabetes. Our previous cross-sectional study proposed an HbA1c cutoff point of 6.3% to diagnose diabetes and 5.9% to diagnose prediabetes in Chinese adults [10]. To evaluate whether HbA1c might be an optimal predictor for progression to diabetes applicable to Chinese adults, we conducted this retrospective cohort study.

Recent longitudinal studies using HbA1c to predict future diabetes risks have yielded various results [16–21]. For example, a US study reported that elevated HbA1c was associated with an increased likelihood of diabetes in older adults [16], which was also confirmed in Indian [17] and Caucasian adults [18]. Studies in Japan [9], Korea [19], and Spain [20] consistently reported that the predictive value of progression to diabetes assessed by HbA1c was similar to that assessed by IFG or IGT alone. In children moreover, a study of American Indians showed that HbA1c was a useful predictor of diabetes risk and might be used to identify prediabetes with the same predictive value as FPG and 2hPG [21]. According to the latest analysis from the Atherosclerosis Risk in Communities (ARIC) study [22], prediabetes defined using the ADA [5] HbA1c cutoff may improve the identification of people at risk for major health complications over the subsequent 10 years compared with prediabetes defined by glucose-based parameters.

Interestingly, we found no significantly higher risk of developing diabetes in nondiabetic subjects whose HbA1c values ranged from 5.7 to 5.8% compared to subjects whose HbA1c values were ≤5.6%, which did not support the ADA-recommended HbA1c value of 5.7% for prediabetes screening in Chinese individuals. Moreover, the ROC curve in the present study showed that an HbA1c value ≥ 5.9% had a higher predictive value of progression to diabetes than an HbA1c value ≥ 5.7%. These present study findings confirmed an HbA1c value of 5.9% as a superior cutoff point for prediabetes. Additionally, we observed that both the FPG concentration of 6.1–6.9 mmol/L and 2hPG concentration of 7.8–11.0 mmol/L had high specificity and low sensitivity for predicting future diabetes risks. In contrast, HbA1c showed high sensitivity and low specificity whether the cutoff point was 5.7% or 5.9%. Therefore, HbA1c and blood glucose used together may efficiently identify subjects who are likely to progress to diabetes and thereby permit early intervention. Although this conclusion is supported by published findings [9, 20, 23, 24], it requires further study in Chinese populations.

Of course, our study had some limitations. First, the possibility of residual confounding cannot be completely eliminated because of the epidemiological nature of our investigation. Moreover, although the HbA1c assay was performed at the same laboratory using standardized methods, we did not examine other blood cell parameters, which might have excluded some conditions that possibly affected the HbA1c results, such as hemoglobinopathies and anemia [25].

In the present study, we aimed to evaluate an indicator to identify subjects at high risk of developing diabetes rather than to detect individuals with prediabetes using different criteria. In this regard, HbA1c performed better than FPG and 2hPG, and an HbA1c cutoff point of 5.9% had a higher predictive value for such risk than the 5.7% value recommended by the ADA. Therefore, we conclude that HbA1c values ≥ 5.9% may be used to more accurately evaluate risk for progression to diabetes among Chinese adults and may identify subjects with prediabetes more reliably.

## Figures and Tables

**Table 1:** Baseline characteristics of individuals with NGT and IGR based on their glycemic status at follow-up.

	NGT at baseline				IGR at baseline			
	Total NGT	Remained as NGT	Progressed to IGR	Progressed to DM	Total IGR	Regressed to NGT	Remained as IGR	Progressed to DM
Numbers (%)	2227	1619 (72.7)	508 (22.8)	100 (4.5)	561	197 (35.1)	254 (45.3)	110 (19.6)
Male, *n* (%)	698	462 (66.2)	194 (27.8)	42 (6.0)	189	64 (33.9)	84 (44.4)	41 (21.7)
Female, *n* (%)	1529	1157 (75.7)	314 (20.5)	58 (3.8)	372	133 (35.8)	170 (45.7)	69 (18.5)
Age (years)	56.9 ± 8.9	56 ± 8.8	59.1 ± 8.7^a^	60.6 ± 7.7^a^	59.75 ± 9.0	57.7 ± 9.1	60.3 ± 8.4^c^	62.0 ± 9.5^c^
BMI (kg/m^2^)	25.9 ± 3.3	25.7 ± 3.3	26.4 ± 3.3^a^	26.5 ± 3.9^a^	26.4 ± 3.3	26.2 ± 3.3	26.4 ± 3.1	26.6 ± 3.8
Waist size, male (cm)	89.6 ± 9.1	88.9 ± 9.1	91.6 ± 9.3^a^	91.8 ± 7.1^a^	90.2 ± 8.9	89.0 ± 9.7	91.0 ± 8.4	90.4 ± 8.7
Waist size, female (cm)	83.5 ± 9.7	82.9 ± 9.6	85.1 ± 9.9^a^	86.5 ± 10.2^a^	86.2 ± 9.6	85.4 ± 10.3	86.4 ± 9.2	87.3 ± 9.3
Systolic BP (mmHg)	138.1 ± 20.1	136.6 ± 20.1	142.1 ± 18.9^a^	141 ± 22.7^a^	140.7 ± 19.9	138.7 ± 18.8	142.3 ± 20.5	140.7 ± 20.3
Diastolic BP (mmHg)	80.1 ± 11.8	79.7 ± 11.8	81.5 ± 11.8^a^	79.9 ± 11.9	82.2 ± 11.5	82.7 ± 11.4	82.6 ± 12.0	80.3 ± 10.6
Heart rate (beats/min)	77.5 ± 10.6	77.1 ± 10.2	78.5 ± 11.3^a^	79.1 ± 12.2	79.0 ± 11.0	79.8 ± 11.3	78.6 ± 11.4	78.4 ± 9.3
FPG (mmol/L)	5.2 ± 0.4	5.1 ± 0.4	5.3 ± 0.4^a^	5.4 ± 0.4^ab^	6.1 ± 0.5	6.0 ± 0.5	6.1 ± 0.5	6.2 ± 0.4^cd^
2hPG (mmol/L)	5.3 ± 1.0	5.2 ± 0.9	5.6 ± 1.0^a^	5.7 ± 1.0^a^	7.7 ± 1.6	7.4 ± 1.6	7.6 ± 1.6	8.2 ± 1.6^cd^
HbA1c (%)	5.7 ± 0.4	5.7 ± 0.3	5.8 ± 0.4^a^	6.1 ± 0.6^ab^	6.0 ± 0.5	5.8 ± 0.4	6.1 ± 0.4^c^	6.4 ± 0.5^cd^
Serum triglycerides (mmol/L)	1.4 ± 0.9	1.4 ± 0.9	1.6 ± 0.9^a^	1.5 ± 0.9	1.7 ± 1.2	1.6 ± 0.9	1.9 ± 1.5^c^	1.8 ± 1.0
Serum cholesterol (mmol/L)	5.3 ± 0.9	5.3 ± 0.9	5.4 ± 1.0^a^	5.3 ± 0.8	5.5 ± 0.9	5.4 ± 0.9	5.6 ± 1.0	5.6 ± 0.9
HDL cholesterol (mmol/L)	1.5 ± 0.3	1.5 ± 0.3	1.4 ± 0.3^a^	1.4 ± 0.3^a^	1.4 ± 0.5	1.5 ± 0.3	1.4 ± 0.3^c^	1.4 ± 0.3^c^
LDL cholesterol (mmol/L)	3.1 ± 0.8	3.1 ± 0.7	3.2 ± 0.8^a^	3.1 ± 0.6	3.3 ± 0.8	3.2 ± 0.8	3.3 ± 0.8	3.3 ± 0.9
Serum Cr (*μ*mol/L)	64.3 ± 10.0	63.7 ± 9.9	65.7 ± 10.0^a^	66.7 ± 11.5^a^	66.3 ± 11.5	66.2 ± 9.9	65.9 ± 11.6	67.6 ± 13.7
ALT (U/L)	12.3 ± 8.3	12.0 ± 7.5	12.5 ± 7.1	15.6 ± 8.7^ab^	12.2 ± 7.7	11.5 ± 6.7	12.7 ± 8.5	12.6 ± 7.3
AST (U/L)	19.8 ± 7.5	19.4 ± 6.3	20.2 ± 8.0	22.5 ± 7.0^ab^	20.0 ± 7.0	19.3 ± 6.3	20.4 ± 7.5	20.4 ± 7.3
GGT (U/L)	24.9 ± 19.7	24.1 ± 20.1	26.8 ± 18.0^a^	26.9 ± 19.2	30.8 ± 24.1	28.7 ± 23.4	28.4 ± 16.6	40.3 ± 25.2^cd^

Values are presented as the mean ± SD for continuous variables and *n* (%) for proportions. ^a^*p* < 0.05 compared with individuals who remained as NGT; ^b^*p* < 0.05 compared to subjects who progressed to IGR; ^c^*p* < 0.05 compared to individuals who regressed to NGT; ^d^*p* < 0.05 compared with individuals who remained as IGR.

**Table 2:** Incidence rates of diabetes among glucose regulation subgroups after 3 years of follow-up.

	Number	Number who progressed to diabetes	Detection incidence	RR	*p* value	Annual incidence
Various glucose tolerances						
NGT^∗^	2227	100	4.5%	1		1.5%
IGR	561	110	19.6%	5.188 (3.885–6.928)	<0.001	6.5%
i-IFG	245	38	15.5%	3.905 (2.618–5.824)	<0.001	5.2%
i-IGT	179	28	15.6%	3.944 (2.514–6.188)	<0.001	5.2%
c-IGR	137	44	32.1%	10.063 (6.673–15.177)	<0.001	10.7%
Various HbA1c levels						
≤5.6^∗^	986	29	2.9%	1		1.0%
5.7–5.8	557	19	3.4%	1.165 (0.647–2.098)	0.610	1.1%
5.9–6.2	813	59	7.3%	2.582 (1.639–4.069)	<0.001	2.4%
6.3–6.4	223	33	14.8%	5.732 (3.399–9.666)	<0.001	4.9%
≥6.5	209	70	33.5%	16.619 (10.408–26.536)	<0.001	11.2%
Total subjects	2778	210	7.6%			2.5%

^∗^Reference group for RR analysis from the chi-square test.

**Table 3:** Predictive value of the diabetes incidence after 3 years of follow-up according to the different IGR definitions at baseline.

	Sensitivity	Specificity	Positive predictive value	Negative predictive value	Positive likelihood ratio	Negative likelihood ratio	Youden index
FPG 6.1–6.9 mmol/L	0.390	0.884	0.215	0.947	3.362	0.690	0.274
2hPG 7.8–11.0 mmol/L	0.343	0.905	0.231	0.944	3.611	0.726	0.248
HbA1c ≥ 5.7%	0.862	0.371	0.100	0.971	1.370	0.372	0.233
HbA1c ≥ 5.9%	0.771	0.580	0.130	0.969	1.836	0.395	0.351
